# Metabolomics and breast cancer: scaling up for robust results

**DOI:** 10.1186/s12916-019-1484-5

**Published:** 2020-01-31

**Authors:** Steven C. Moore

**Affiliations:** Metabolic Epidemiology Branch, Division of Epidemiology and Genetics, National Cancer Institute, National Institutes of Health, 9609 Medical Center Dr, Bethesda, MD 20892 USA

**Keywords:** Metabolomics, Metabolism, Lipid, Breast cancer, Reproducibility

## Background

Breast cancer is the most commonly diagnosed cancer among women, with more than 2 million new cases diagnosed in 2018 [1], and rapidly rising global incidence. Though breast cancer has been extensively studied, established risk factors explain only half of breast cancer incidence [2], and few are modifiable. Identifying further risk factors is urgently needed to inform cancer control strategies to slow the rise in the rate of breast cancer. New ‘omics-based approaches to exposure measurement may enable hundreds to thousands of novel exposures to be evaluated simultaneously, often in pathways that would otherwise be unstudied.

## Metabolism and risk of breast cancer

In a recent article, His et al. used metabolomics to examine levels of 127 circulating metabolites and their association with breast cancer risk [3]. Metabolomics involves measuring hundreds of metabolites from human metabolic processes, such as food digestion and energy homeostasis. Metabolism is a prime target for research since many aspects can be modified through diet, weight control, or physical activity. Strong evidence implicates obesity and physical inactivity in breast cancer risk [4].

His et al. found that levels of acylcarnitine C2 are positively associated with breast cancer risk, and levels of phosphatidylcholine (PC) ae C36:3 are inversely associated with risk in the overall population. The findings for acylcarnitine C2 are particularly intriguing. Acylcarnitine C2 facilitates the transport of fatty acids into the mitochondria. Higher levels are a marker for lipid oversupply and upregulated fatty acid oxidation [5]. In cancer cell biology, lipid oversupply is thought to enhance cancer cell proliferation by providing the raw materials needed to generate new cells [6]. Though speculative, it is suggested that chronic lipid oversupply increases breast cancer risk, perhaps by supplying energy and nutrients to growing tumors.

The inverse association between PC ae C36:3 and risk is more challenging to interpret. This measure is a sum of multiple phosphatidylcholines, and little is known about individual phosphatidylcholines and their relation to health. Another finding of His et al. was that metabolite associations with risk were greater in number and magnitude among non-hormone users (70% of the cases). As the authors note, this pattern echoes that of biomarker studies outside of the area of metabolomics. Non-hormone users may be a special population of interest for future metabolomics and breast cancer studies.

The findings of the study by His et al. are biologically intriguing and potentially important, but we must now acknowledge a complicating fact: their findings do not replicate those of preceding prospective studies on metabolomics and breast cancer [7–9] – and not likely because of deficient study design. With 1624 breast cancer cases, the study by His et al. is by far the largest on this topic (with 1000 more cases than the next largest study [9]) and the Biocrates assay was highly reliable. Statistical analysis was careful, and the authors conclusions were conservative. All things considered, the study by His et al. may, in fact, be the most methodologically sound study to date on this topic.

## Heterogeneity among study results

The lack of replication, then, may reflect broader issues in metabolomics and breast cancer research. One pressing issue is that metabolomics platforms measure different sets of metabolites, which foils attempts to replicate results from study to study. For example, a prior prospective study using the Metabolon platform found that specific branched-chain amino acid byproducts and sex steroid hormone metabolites were associated with breast cancer risk [9]. However, His et al. could not evaluate these associations because the Biocrates assay did not measure the relevant metabolites. For researchers to uncover reproducible associations, it is essential that metabolomics platforms improve in terms of coverage and their overlap with one another. It may also be valuable for researchers to develop methods for imputing missing metabolites, as done in genome-wide association studies, to improve harmonization across studies.

A second important issue is that, typically, sample sizes in metabolomics–breast cancer studies have been small (i.e., 621 or fewer cases), possibly resulting in false positives and failures to replicate. Several of the metabolites measured by His et al. were previously associated with breast cancer risk: valine [8, 9], glutamate [9], lysine [8], arginine [8], glutamine [8], creatinine [8], phosphatidylcholine acyl-alkyl C30:0 (PC ae C30:0) [7], and lysophosphatidylcholine acyl C18:0 (lysoPC a C18:0) [7]. In His et al., though, the associations did not replicate, highlighting that initial studies perhaps needed to be larger to yield robust results.

These failures to replicate may surprise researchers studying other diseases, for which metabolomics findings have generally been able to be replicated. In diabetes research, for example, a metabolic profiling study published in 2011 identified associations between branched-chain and aromatic amino acids and diabetes risk [10], and these findings have been replicated many times since. If diabetes findings replicate with ease, why not those for breast cancer? Possibly, metabolic factors just have comparatively modest associations with breast cancer risk. For instance, acylcarnitine C2 in His et al. is associated with a 15% increase in breast cancer risk per standard deviation increment, whereas several metabolites are associated with 50–100% increases in diabetes risk per standard deviation increment [10].

Clarifying breast cancer risk factors is undoubtedly important, but if associations are of modest to moderate magnitude, then special care must be taken with study design. Genomics researchers learned this lesson the hard way after a “lost decade” of irreproducible studies on common genetic variants – a problem they ultimately resolved by scaling up studies, imputing variants across studies, and pooling data into massive consortium-based analyses. Metabolomics researchers who are focused on breast cancer – or any cancer for that matter – should heed these lessons.

## Conclusion

In the largest prospective study of metabolomics and cancer to date, His et al. make a key advance in the hunt for metabolic breast cancer risk factors. Their findings suggest new etiologic clues and provide a robust foundation upon which future studies can build. Going forward, researchers should consider with care how best to build upon this foundation.

## Data Availability

Not applicable.

## References

[1] Bray F, Ferlay J, Soerjomataram I, Siegel RL, Torre LA, Jemal A (2018). Global cancer statistics 2018: GLOBOCAN estimates of incidence and mortality worldwide for 36 cancers in 185 countries. CA Cancer J Clin.

[2] Collaborative Group on Hormonal Factors in Breast Cancer (2002). Breast cancer and breastfeeding: collaborative reanalysis of individual data from 47 epidemiological studies in 30 countries, including 50302 women with breast cancer and 96973 women without the disease. Lancet..

[3] His M, Viallon V, Dossus L, Gicquiau A, Achaintre D, Scalbert A (2019). Prospective analysis of circulating metabolites and breast cancer in EPIC. BMC Med.

[4] World Cancer Research Fund, American Institute for Cancer Research (2018). Diet, nutrition, physical activity and breast cancer: a global perspective. Continuous Update Project Expert Report.

[5] Bene J, Hadzsiev K, Melegh B (2018). Role of carnitine and its derivatives in the development and management of type 2 diabetes. Nutr Diabetes.

[6] Carracedo A, Cantley LC, Pandolfi PP (2013). Cancer metabolism: fatty acid oxidation in the limelight. Nat Rev Cancer.

[7] Kühn T, Floegel A, Sookthai D, Johnson T, Rolle-Kampczyk U, Otto W (2016). Higher plasma levels of lysophosphatidylcholine 18:0 are related to a lower risk of common cancers in a prospective metabolomics study. BMC Med.

[8] Lécuyer L, Victor Bala A, Deschasaux M, Bouchemal N, Nawfal Triba M, Vasson MP (2018). NMR metabolomic signatures reveal predictive plasma metabolites associated with long-term risk of developing breast cancer. Int J Epidemiol.

[9] Moore SC, Playdon MC, Sampson JN, Hoover RN, Trabert B, Matthews CE (2018). A metabolomics analysis of body mass index and postmenopausal breast cancer risk. J Natl Cancer Inst.

[10] Wang TJ, Larson MG, Vasan RS, Cheng S, Rhee EP, McCabe E (2011). Metabolite profiles and the risk of developing diabetes. Nat Med.

